# Supplementary material for: Sugar-based synthesis of an enantiomorphically pure zeolite
Source: Nat Commun. 2024 Jun 21;15:5298. doi: 10.1038/s41467-024-49659-2 (PMC11192950; doi:10.1038/s41467-024-49659-2)
Supplement: Supplementary file 1 — Supplementary Information [file 41467_2024_49659_MOESM1_ESM.pdf]

# Supplementary Information

## Sugar-based synthesis of an enantiomorphically pure zeolite

Andrés Sala[1], José L. Jordá[1], German Sastre[1], Antonio L. Llamas-Saiz[2], Fernando Rey[1]*, Susana Valencia[1]*

Corresponding authors: frey@itq.upv.es; svalenci@itq.upv.es

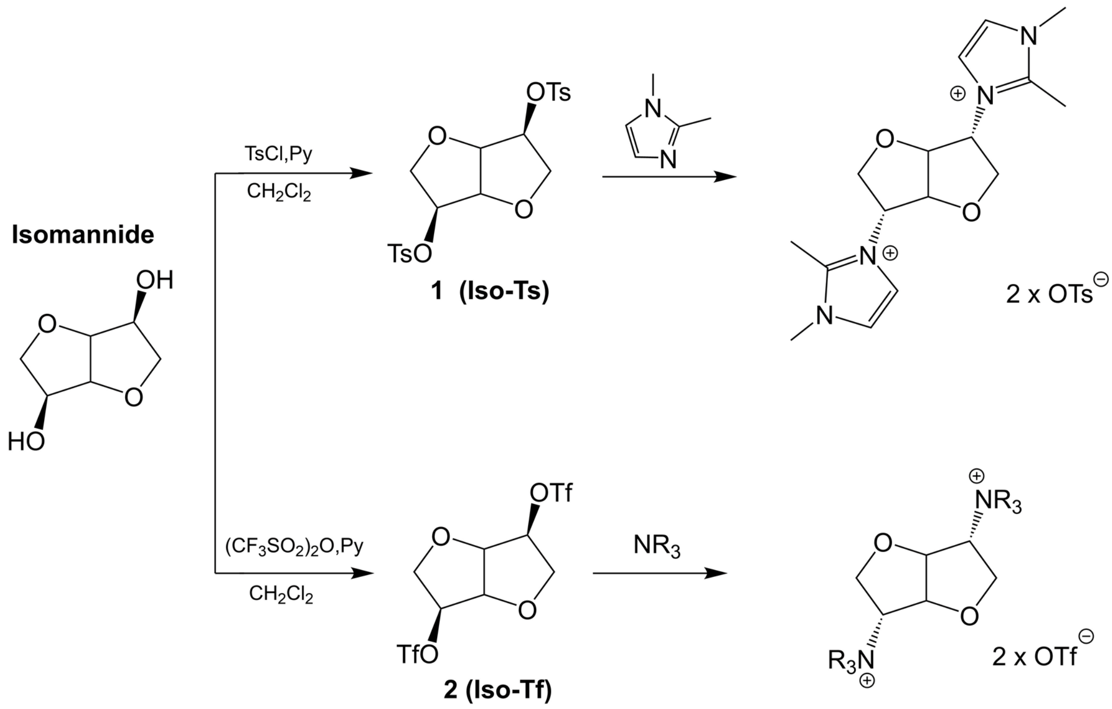


**Supplementary Figure 1.**
General organic synthesis route followed to obtain the organic structure directing agents.

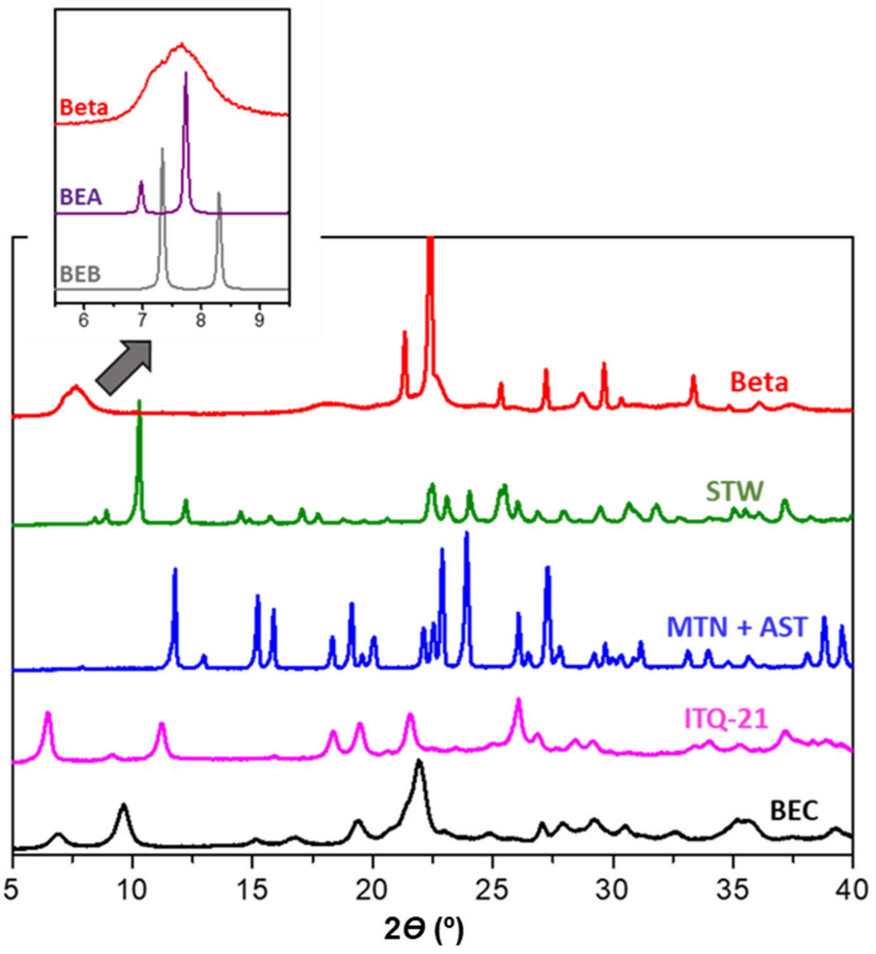


**Supplementary Figure 2.**

Typical X-ray diffraction patterns of the zeolites obtained using sugar derived OSDAs (specific conditions in Table 1). The inset shows that there is no a clear evidence of preferential crystallization of any polymorph of zeolite Beta. Source data are provided as a Source Data file.

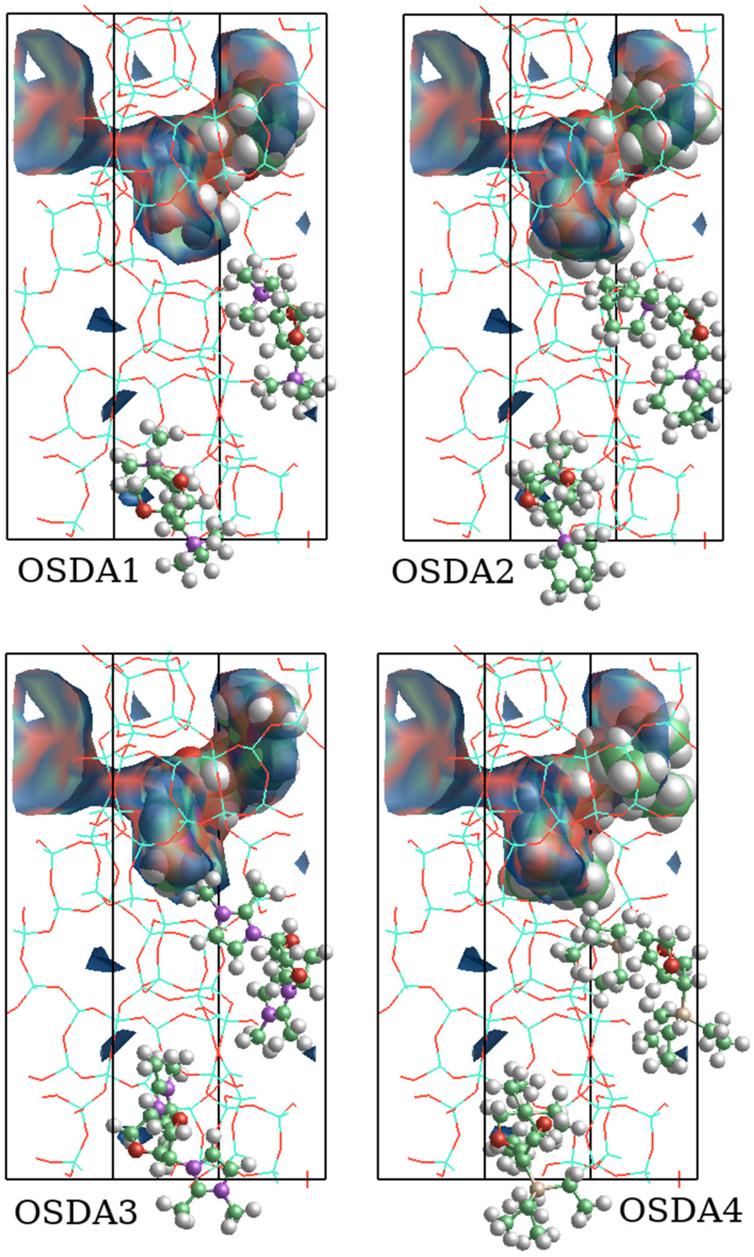


**Supplementary Figure 3.**

Optimized geometries of OSDAs in Figure 1 in S-STW zeolite ($P6_122$ space group) using the available structural data. View of 1×1×1 STW unit cell showing 3 OSDA molecules, where the upper OSDA has been highlighted with larger balls as well as its corresponding micropore: the microporous surface of the upper OSDA has been made opaque in order to show only those OSDA atoms protruding outside the micropore, which contribute negatively to zeo-OSDA stabilization. OSDA3 is the one with less number of atoms protruding outside the micropore.

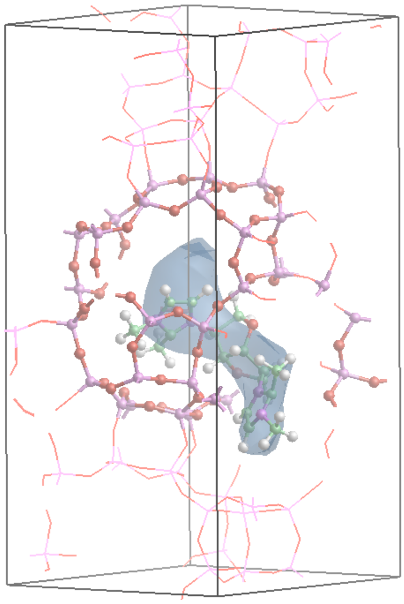

**Supplementary Figure 4.**

Optimal OSDA3 location in R-STW: Illustrating the poor alignment within its helicoidal channel. Our Monte Carlo algorithm, requiring 10 times more cycles (120000), reveals that the curvature of OSDA3 does not align well with the helicoidal channel of R-STW and only one OSDA3 molecule could be accommodated within the unit cell of R-STW, contrasting with S-STW where three OSDA3 molecules could be readily allocated within its micropore system using the same Monte Carlo algorithm.

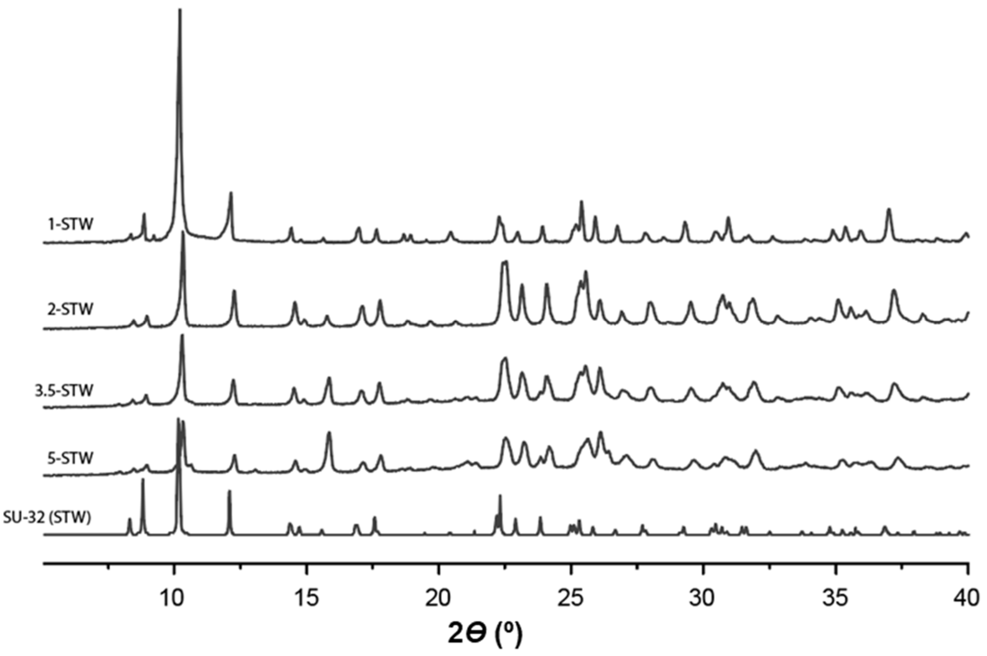


**Supplementary Figure 5.**

X-ray diffraction patterns of the as-made STW zeolites synthesized with OSDA3 from gels of different Si/Ge ratio (labelled in each pattern). XRD pattern of reference STW is shown for comparison as appears in the Database of Zeolite Structures of the Structure Commission of the IZA and correspond to the pure Ge-STW[9]. Source data are provided as a Source Data file.

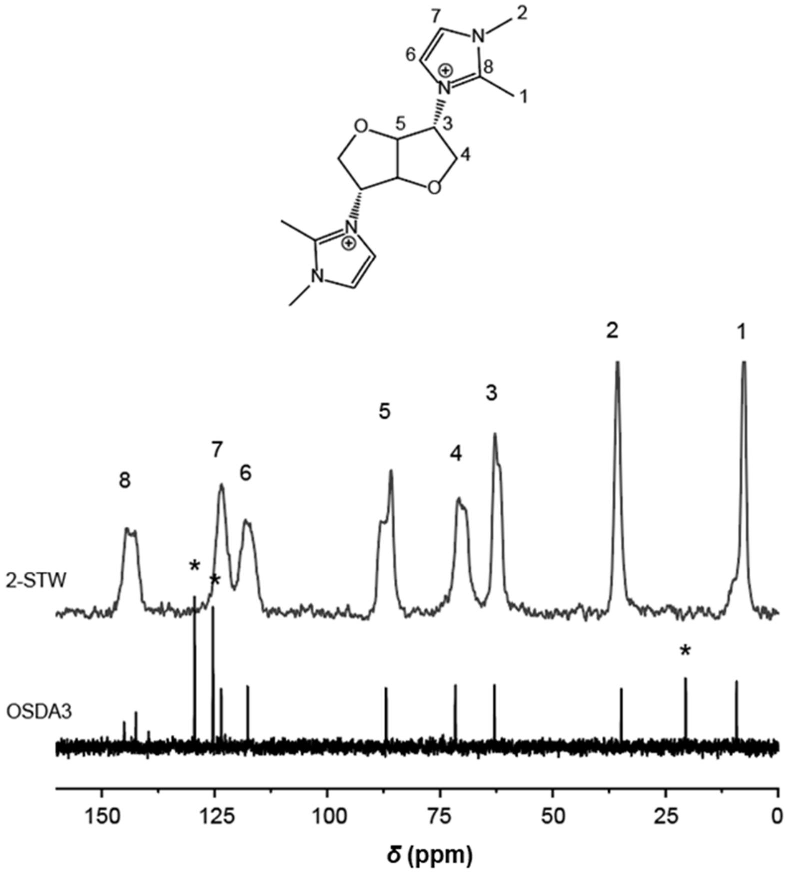


**Supplementary Figure 6.**

$^{13}$C-NMR spectrum of OSDA3 in $D_2O$ (bottom) and solid state $^{13}$C-CP-SS-NMR spectrum of 2-STW zeolite (top). Source data are provided as a Source Data file.

The observed splitting of the resonances in the $^{13}$C-CP-SS-NMR spectrum of 2-STW zeolite may be attributed to the presence of four different configuration of the encapsulated OSDA within the pore system of the zeolite.

*corresponds to the signals of the tosylate anion ($TsO^-$) not present in the zeolite.

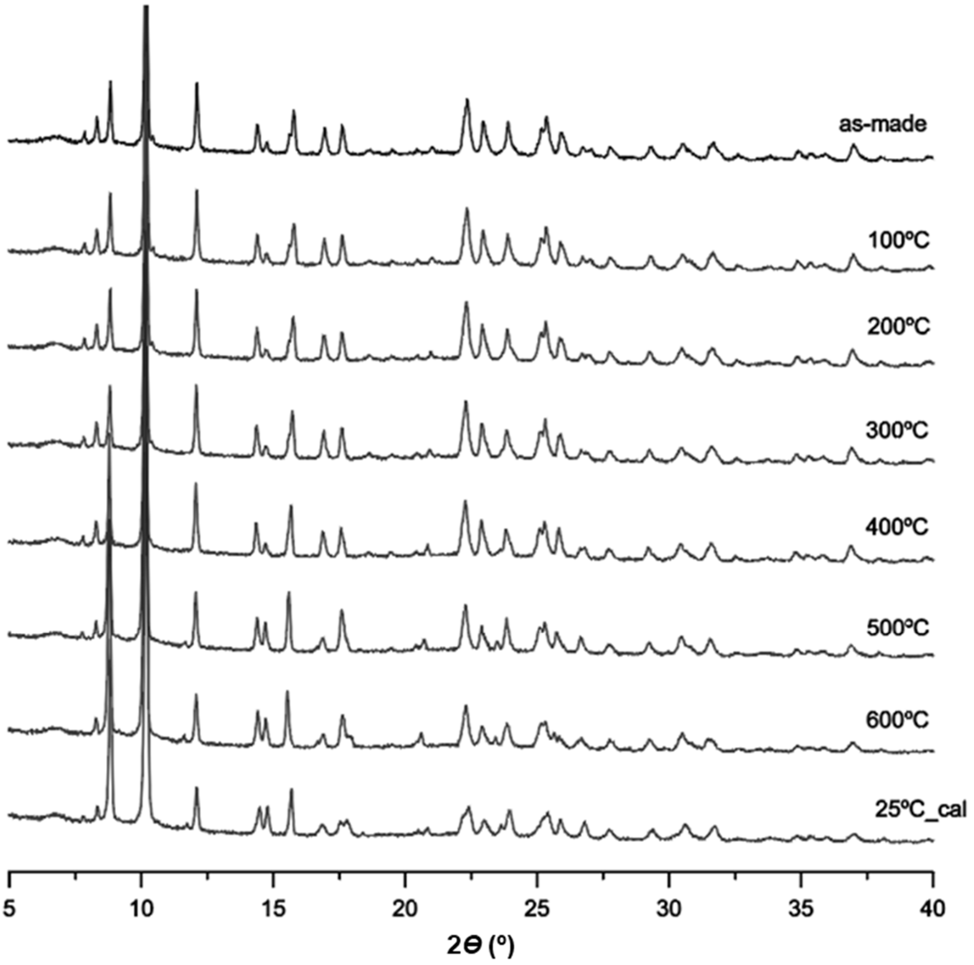


**Supplementary Figure 7.**

X-ray diffraction patterns of the in-situ calcination of 2-STW zeolite under dry air. From top to bottom the temperature was increased step by step up to 600 ºC. XRD at the bottom corresponds to the sample calcined at 600 ºC and measured at room temperature. Source data are provided as a Source Data file.

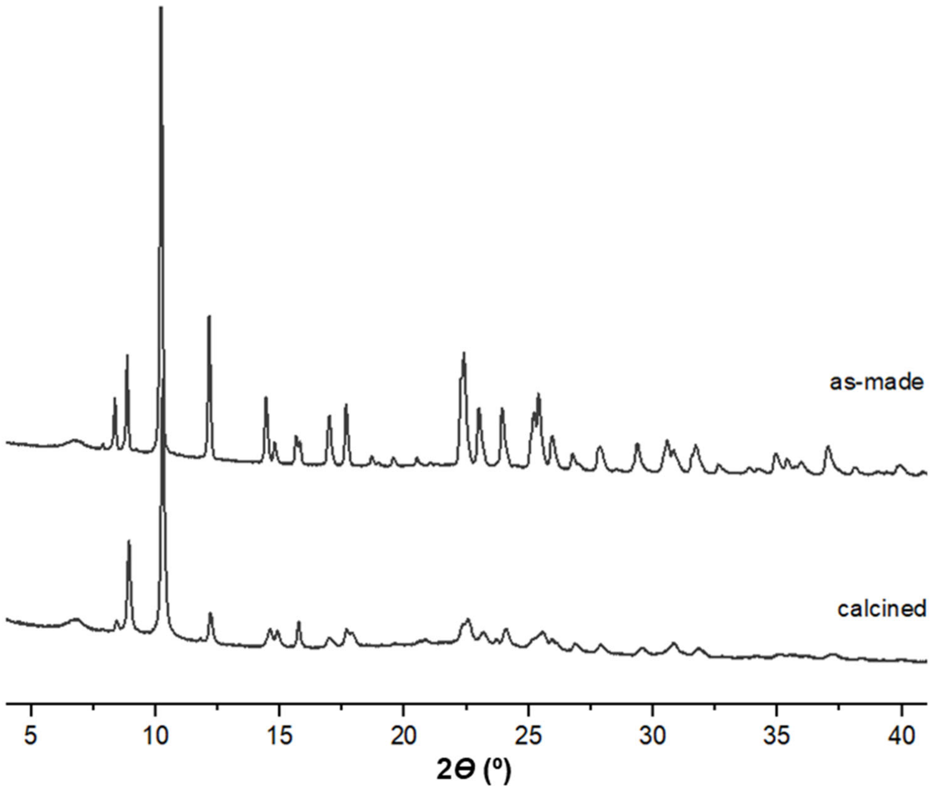


**Supplementary Figure 8.**
Comparison of the X-ray diffraction patterns of as-made and calcined 2-STW zeolite (calcination at 600 ºC under dry air).

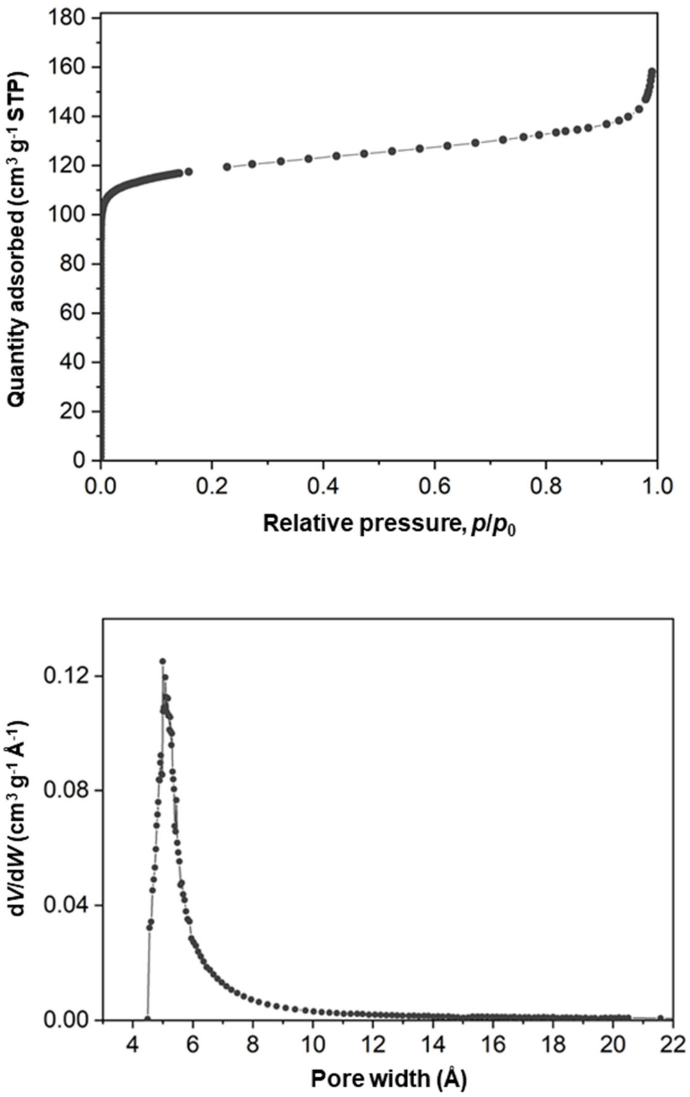


**Supplementary Figure 9.**

Argon adsorption isotherm at 87 K of calcined 2-STW zeolite (top), and the corresponding Horvath-Kawazoe differential pore-size distribution curve (bottom). Source data are provided as a Source Data file.

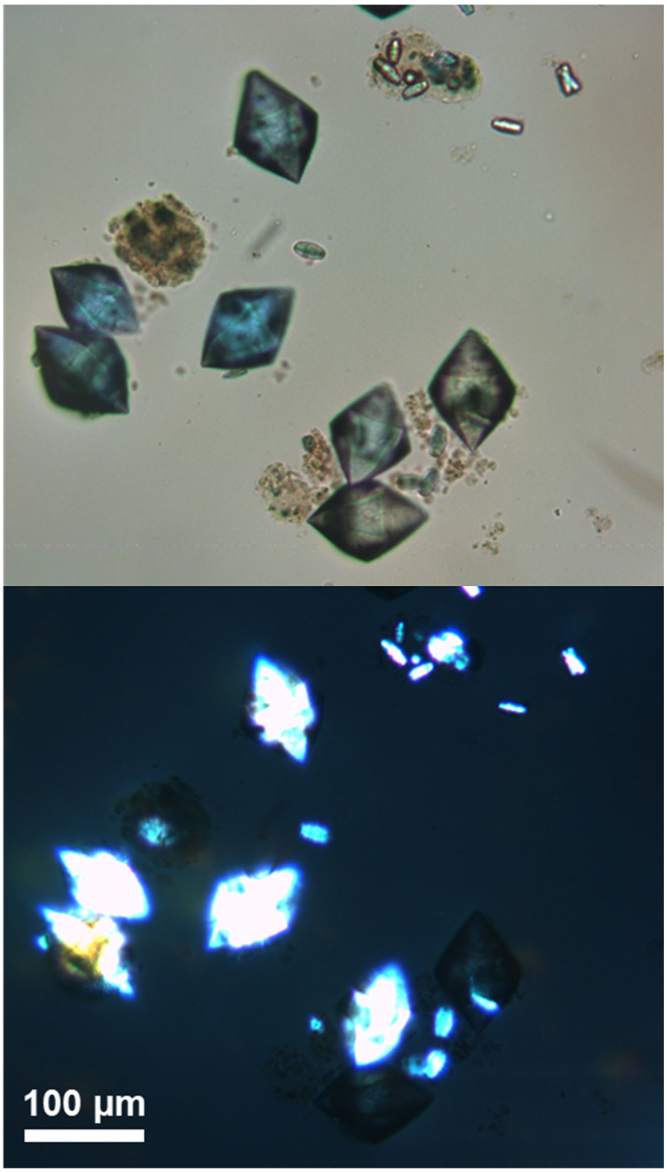


**Supplementary Figure 10.**

Polarized optical microscopy images of as-made 2-STW zeolite under light field (top) and dark field (bottom). Rod-like crystals are minor impurities of quartz-like $GeO_2$ in the studied sample.

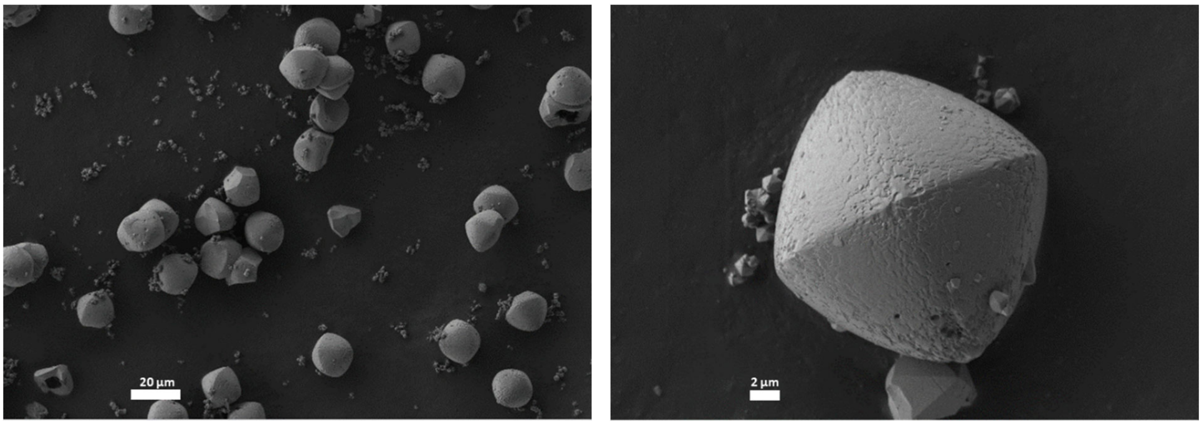


**Supplementary Figure 11.**

Field emission scanning electron microscopy images of racemic 2-STW sample in the as-made form at different magnifications.

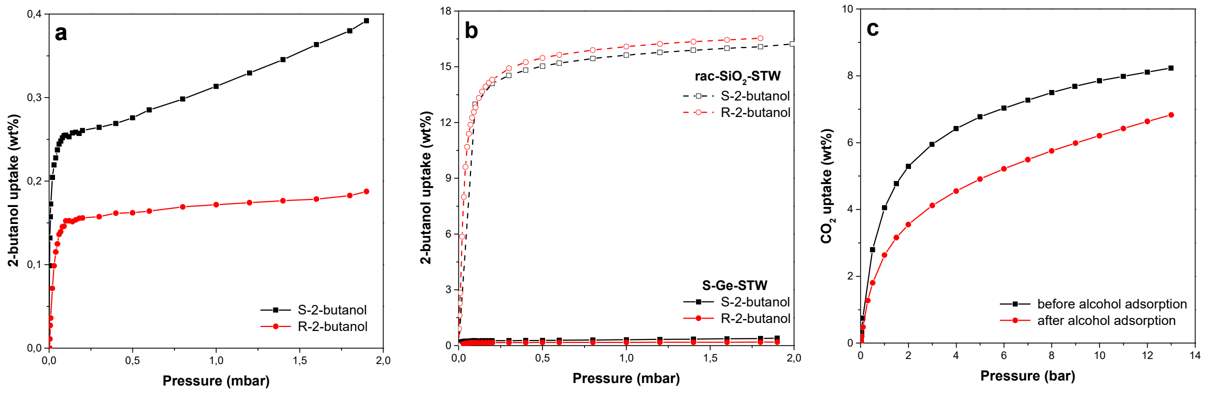


**Supplementary Figure 12.**

(a) Non-equilibrated adsorption isotherms of R and S isomers of 2-butanol at 10ºC on 2-STW sample (S-enantiomorph). (b) Adsorption isotherms of R and S isomers of 2-butanol at 10ºC on a pure silica STW material (racemic) compared to those obtained on 2-STW sample. The racemic pure silica STW sample shows a crystal size of around 0.5 to 5 microns, which is between 10 and 100 times smaller than enantiomorphically pure 2-STW sample. (c) $CO_2$ adsorption isotherms at 25ºC on 2-STW sample before and after 2-butanol adsorption experiments. Source data are provided as a Source Data file.

Despite the strong diffusion limitations of S-butanol and R-butanol to access the microporosity of the enantiomerically pure Ge-containing S-STW zeolite (2-STW), it is remarkable that the adsorption capacity of S-2-butanol is nearly double that of R-2-butanol, as shown in Figure a, suggesting the preferential adsorption of the S over the R-enantiomer. However, it is important to highlight that the expected adsorption capacities, as evidenced in the case of pure silica racemic STW (Figure b), are approximately 15 wt.%, whilst on Ge-S-STW material does not reach 1 wt.%. This introduces important uncertainties on the 2-butanol adsorption results. Furthermore, the notably low adsorption capacities of 2-butanol isomers cannot be attributed to the loss of crystallinity of the Ge-containing S-STW, as approximately 80 % of its microporosity remains intact after alcohol adsorption experiments, as deduced from the $CO_2$ isotherms (Figure c).

**Supplementary Table 1.**

Calculated S-STW-OSDA van der Waals energies (Supplementary Fig. 3), using the available structural data, for full packing of the OSDA molecules of Fig. 1.

| **ch-OSDA** | $E_{zeo\text{-}OSDA}$ (kJ per OSDA) |
|---|---|
| OSDA1 | +50.1 |
| OSDA2 | +35.4 |
| OSDA3 | -178.3 |
| OSDA4 | +78.0 |

**Supplementary Table 2.**
Chemical compositions of selected STW samples.

| Sample | Si/Ge (gel) | (Si+Ge)/Al (gel) | Experimental weight contents (wt %) | | | Experimental molar ratios | | |
|---|---|---|---|---|---|---|---|---|
| | | | $SiO_2$ | $GeO_2$ | $Al_2O_3$ | Si/Ge | (Si+Ge)/Al | Si/Al |
| **2-STW*** | 2 | - | 32.9 | 45.4 | - | 1.3 | - | - |
| **3.5-STW** | 3.5 | - | 47.3 | 32.3 | - | 2.6 | - | - |
| **5-STW** | 5 | - | 57.9 | 27.9 | - | 4.4 | - | - |
| **2-STW-100** | 2 | 100 | 29.8 | 46.9 | 0.2 | 1.1 | 245 | 129 |
| **2-STW-50** | 2 | 50 | 33.6 | 45.8 | 0.4 | 1.2 | 130 | 74 |
| **2-STW-25** | 2 | 25 | 31.1 | 41.9 | 1.1 | 1.3 | 43 | 25 |
| **2-STW-12** | 2 | 12 | 31.7 | 33.9 | 3.0 | 1.6 | 14 | 9 |

* The C and N contents were determined by elemental analysis of sample 2-STW, resulting in 9.4 wt.% and 2.5 wt.% respectively. The F content was calculated from the $^{19}F$ MAS-NMR spectrum of the weighed sample 2-STW, yielding 1.85 wt.%. The final experimental chemical composition of sample 2-STW was as follows: $C_{47.8}H_{72}N_{10.9}F_{5.9}Si_{33.5}Ge_{26.5}O_{126}$. The H and O contents were not directly determined; the reported values are those anticipated from the assumption of 3 molecules of OSDA-3 per unit cell.

**Supplementary Table 3.**

Textural properties of calcined 2-STW sample obtained from the $N_2$ and Ar adsorption isotherms and comparison with HPM-1 zeolite.

| Zeolite | $S_{BET}$[1] ($m^2\ g^{-1}$) | *t*-plot micropore volume ($cm^3\ g^{-1}$) | Pore diameter (Å) |
|---|---|---|---|
| **2-STW** | 449 | 0.17 | 5 |
| **Si-STW**[2] | 597 | 0.23 | 5 |
| **HPM-1**[3] | 630 | 0.24 | 5 |

[1] $S_{BET}$ stands for apparent BET Surface Area
[2] Data of 2-STW sample recalculated for a hypothetical pure silica STW.
[3] Synthesized according to ref. 31.

**Supplementary Table 4.**

Summary of the crystallographic information of 30 crystals of different STW samples obtained using chiral OSDA3, compiled from the individual single crystal X-ray diffraction analyses.

| Sample | | Crystal | Space group[a] | *a* (Å) | *c* (Å) | Flack parameter[b] |
|---|---|---|---|---|---|---|
| **2-STWb** | 1 | 18RGA008 | $P6_122$ (S) | 12.1912(16) | 30.031(5) | -0.027(14) |
| | 2 | 18RGA008B | $P6_122$ (S) | 12.222(2) | 30.161(9) | -0.02(9) |
| | 3 | 18RGA008C | $P6_122$ (S) | 12.2031(11) | 30.095(4) | -0.09(8) |
| | 4 | 18RGA008D | $P6_122$ (S) | 12.2077(9) | 30.130(3) | -0.10(8) |
| | 5 | 18RGA008E | $P6_122$ (S) | 12.2093(9) | 30.132(4) | -0.05(5) |
| | 6 | 18RGA008F[*] | $P6_122$ (S) | 12.2110(8) | 30.118(3) | 0.02(6) |
| | 7 | 18RGA008G | $P6_122$ (S) | 12.2031(13) | 30.095(5) | -0.11(8) |
| | 8 | 18RGA008H | $P6_122$ (S) | 12.2031(11) | 30.118(4) | 0.01(6) |
| | 9 | 18RGA008I | $P6_122$ (S) | 12.2074(19) | 30.115(7) | -0.11(8) |
| | 10 | 18RGA008J | $P6_122$ (S) | 12.2034(10) | 30.107(4) | -0.03(10) |
| | | average | 100% (S) | 12.2061(4) | 30.1102(17) | - |
| **2-STWa** | 11 | 19RGA003 | $P6_122$ (S) | 12.1504(4) | 29.9991(8) | 0.01(2) |
| | 12 | 19RGA003B | $P6_122$ (S) | 12.1497(2) | 30.0043(11) | 0.03(3) |
| | 13 | 19RGA003C | $P6_122$ (S) | 12.1511(3) | 30.0081(15) | -0.02(2) |
| | 14 | 19RGA003D | $P6_122$ (S) | 12.1481(3) | 30.0007(9) | 0.05(3) |
| | 15 | 19RGA003E | $P6_122$ (S) | 12.1514(4) | 30.0040(8) | 0.03(3) |
| | 16 | 19RGA003F | $P6_122$ (S) | 12.1481(2) | 29.9973(11) | 0.00(3) |
| | 17 | 19RGA003G | $P6_122$ (S) | 12.1494(2) | 30.0017(14) | 0.00(3) |
| | 18 | 19RGA003H | $P6_122$ (S) | 12.1465(3) | 29.9949(12) | -0.02(3) |
| | 19 | 19RGA003I | $P6_122$ (S) | 12.1554(4) | 30.0208(10) | 0.000(9) |
| | 20 | 19RGA003J | $P6_122$ (S) | 12.1481(2) | 29.9975(11) | -0.04(3) |
| | | average | 100% (S) | 12.1498(1) | 30.0028(4) | - |
| **2-STW-25** | 21 | 21RGA002 | $P6_122$ (S) | 12.1686(2) | 30.0474(9) | -0.02(2) |
| | 22 | 21RGA002B | $P6_122$ (S) | 12.1763(2) | 30.0614(8) | 0.012(7) |
| | 23 | 21RGA002C | $P6_122$ (S) | 12.16670(14) | 30.0448(7) | -0.02(2) |
| | 24 | 21RGA002D | $P6_122$ (S) | 12.17020(14) | 30.0525(8) | -0.03(2) |
| | 25 | 21RGA002E | $P6_122$ (S) | 12.1693(2) | 30.0487(8) | -0.01(2) |
| | 26 | 21RGA002F | $P6_122$ (S) | 12.1708(3) | 30.0623(8) | -0.02(2) |
| | 27 | 21RGA002G | $P6_122$ (S) | 12.1744(3) | 30.0663(10) | 0.012(7) |
| | 28 | 21RGA002H | $P6_122$ (S) | 12.1724(3) | 30.0541(12) | 0.01(2) |
| | 29 | 21RGA002I | $P6_122$ (S) | 12.1690(2) | 30.0473(8) | -0.02(2) |
| | 30 | 21RGA002J | $P6_122$ (S) | 12.17090(14) | 30.0540(7) | 0.00(2) |
| | | average | 100% (S) | 12.1709(1) | 30.0539(3) | - |

[a] Hexagonal crystal system: $a = b$, $\alpha = \beta = 90^o$, $\gamma = 120^o$. [b] The Flack parameter is determined by quotients $\{(I^+)-(I^-)]/[(I^+)+(I^-)\}$[18,19]. Values close to 0 indicate correct enantiomorph and values close to 1 the opposite.

*Crystal used for refinement and complete structural solution.

**Supplementary Table 5.**

Summary of the crystallographic information of 11 crystals of a STW sample synthesized using a non-chiral OSDA, according to reference 36, compiled from the individual single crystal X-ray diffraction analyses.

| Sample | | Crystal | Space group | *a* (Å) | *c* (Å) | Flack parameter [a] |
|---|---|---|---|---|---|---|
| **2-STWrac** | 1 | 21RGA003 | $P6_122$ (S) | 12.1050(11) | 30.126(5) | 0.03(6) |
| | 2 | 21RGA003A | $P6_122$ (S) | 12.1039(8) | 30.129(3) | -0.02(4) |
| | 3 | 21RGA003B | $P6_522$ (R) | 12.1030(9) | 30.128(4) | -0.01(4) |
| | 4 | 21RGA003C | $P6_122$ (S) | 12.1033(11) | 30.122(5) | -0.02(5) |
| | 5 | 21RGA003D | $P6_522$ (R) | 12.1025(9) | 30.123(4) | -0.01(4) |
| | 6 | 21RGA003E | $P6_122$ (S) | 12.096(3) | 30.142(12) | -0.02(8) |
| | 7 | 21RGA003F | $P6_522$ (R) | 12.1006(13) | 30.119(4) | 0.05(5) |
| | 8 | 21RGA003G | $P6_522$ (R) | 12.1047(8) | 30.132(3) | -0.06(4) |
| | 9 | 21RGA003H | $P6_522$ (R) | 12.0989(9) | 30.119(4) | -0.07(5) |
| | 10 | 21RGA003I | $P6_522$ (R) | 12.117(4) | 30.189(14) | 0.01(4) |
| | 11 | 21RGA003J | $P6_122$ (S) | 12.1036(9) | 30.125(3) | 0.04(5) |
| | | average | 45% (S)<br>55% (R) | 12.1035(6) | 30.1322(21) | - |

[a] Hexagonal crystal system: $a = b$, $\alpha = \beta = 90°$, $\gamma = 120°$. [b] The Flack parameter is determined by quotients $\{(I^+)-(I^-)]/[(I^+)+(I^-)\}$[18,19]. Values close to 0 indicate correct enantiomorph and values close to 1 the opposite.

**Supplementary Table 6.**
Summary of the experimental crystal structure determination for **2-STWb** (crystal 18RGA008F).

*Crystal data*

| | |
|---|---|
| $2(Ge_{2.61}O_{10}Si_{2.39})\cdot 2(C_4H_6NO_{0.50})\cdot F$ | $D_x$ = 2.576 Mg m$^{-3}$ |
| $M_r$ = 1004.26 | Cu $K\alpha$ radiation, $\lambda$ = 1.54178 Å |
| Hexagonal, $P6_122$ | Cell parameters from 9252 reflections |
| $a$ = 12.2039 (1) Å | $\theta$ = 4.2–78.9° |
| $c$ = 30.1128 (7) Å | $\mu$ = 10.02 mm$^{-1}$ |
| $V$ = 3884.00 (11) Å$^3$ | $T$ = 100 K |
| $Z$ = 6 | Prism, clear colourless |
| $F$(000) = 2909 | 0.09 × 0.07 × 0.05 mm$^3$ |

*Data collection*

| | |
|---|---|
| Bruker D8 VENTURE PHOTON-III C14 diffractometer | 2826 independent reflections |
| Radiation source: microfocus sealed tube, Incoatec IµS 3.0 | 2732 reflections with $I > 2\sigma(I)$ |
| Multilayer mirror monochromator | $R_{int}$ = 0.053 |
| Detector resolution: 7.3910 pixels mm$^{-1}$ | $\theta_{max}$ = 79.7°, $\theta_{min}$ = 4.2° |
| $\phi$ or $\omega$ oscillation scans | $h$ = -15→15 |
| Absorption correction: multi-scan *SADABS2016*/2 - Bruker AXS area detector scaling and absorption correction | $k$ = -15→15 |
| $T_{min}$ = 0.55, $T_{max}$ = 0.62 | $l$ = -38→38 |
| 172040 measured reflections | |

*Refinement*

| | |
|---|---|
| Refinement on $F^2$ | Secondary atom site location: difference Fourier map |
| Least-squares matrix: full | Hydrogen site location: inferred from neighbouring sites |
| $R[F^2 > 2\sigma(F^2)]$ = 0.018 | H-atom parameters constrained |
| $wR(F^2)$ = 0.044 | $w = 1/[\sigma^2(F_o^2) + (0.0217P)^2 + 3.935P]$ where $P = (F_o^2 + 2F_c^2)/3$ |
| $S$ = 1.14 | $(\Delta/\sigma)_{max}$ = 0.001 |

| | |
|---|---|
| 2826 reflections | $\Delta\rho_{max}$ = 0.24 e Å$^{-3}$ |
| 403 parameters | $\Delta\rho_{min}$ = -0.31 e Å$^{-3}$ |
| 681 restraints | Absolute structure: Flack x determined using 1040 quotients $[(I^+)-(I^-)]/[(I^+)+(I^-)]$ (Parsons, Flack and Wagner, Acta Cryst. B69 (2013) 249-259). |
| Primary atom site location: dual | Absolute structure parameter: -0.014 (10) |

CCDC-2278869 to CCDC-2278910 contains the supplementary crystallographic data for all the single crystals studied in this paper. The data can be obtained free of charge from The Cambridge Crystallographic Data Centre via www.ccdc.cam.ac.uk/structures.

**Supplementary Table 7.**

Crystallographic parameters of as-made STW refined by PXRD.

| Site | Atom | *x* | *y* | *z* | Occupancy | Uiso | Wyckoff |
|---|---|---|---|---|---|---|---|
| Si1 | Si | 0.7023(4) | 0.3804(5) | 0.03333(15) | 0.632(9) | 0.0413(6) | 12c |
| Ge1 | Ge | 0.7023(4) | 0.3804(5) | 0.03333(15) | 0.473(9) | 0.0413(6) | 12c |
| Si2 | Si | 0.5294(4) | 0.4167(4) | -0.03348(13) | 0.280(7) | 0.0413(6) | 12c |
| Ge2 | Ge | 0.5294(4) | 0.4167(4) | -0.03348(13) | 0.720(7) | 0.0413(6) | 12c |
| Si3 | Si | 0.3036(4) | 0.3949(4) | -0.14947(16) | 0.365(10) | 0.0413(6) | 12c |
| Ge3 | Ge | 0.3036(4) | 0.3949(4) | -0.14947(16) | 0.635(10) | 0.0413(6) | 12c |
| Si4 | Si | 0.2446(4) | 0.3381(4) | -0.04889(16) | 0.457(9) | 0.0413(6) | 12c |
| Ge4 | Ge | 0.2446(4) | 0.3381(4) | -0.04889(16) | 0.543(9) | 0.0413(6) | 12c |
| Si5 | Si | -0.0336(5) | 0.1156(4) | -0.03344(15) | 0.873(8) | 0.0413(6) | 12c |
| Ge5 | Ge | -0.0336(5) | 0.1156(4) | -0.03344(15) | 0.127(8) | 0.0413(6) | 12c |
| O1 | O | -0.087(3) | 0.00000 | 0.00000 | 1.000(8) | 0.063(3) | 6a |
| O2 | O | 0.5727(13) | 0.3394(14) | 0.0032(6) | 1.00000 | 0.063(3) | 12c |
| O3 | O | 0.1195(9) | 0.2139(16) | -0.0242(6) | 1.000(8) | 0.063(3) | 12c |
| O4 | O | -0.0651(15) | 0.0651(15) | -0.0833 | 1.000(8) | 0.063(3) | 6b |
| O5 | O | -0.1041(17) | 0.1970(15) | -0.0214(5) | 1.000(8) | 0.063(3) | 12c |
| O6 | O | 0.663(2) | 0.331(2) | 0.08333 | 1.000(8) | 0.063(3) | 6b |
| O7 | O | 0.3717(8) | 0.3384(18) | -0.0312(7) | 1.000(8) | 0.063(3) | 12c |
| O8 | O | 0.7735(14) | 0.3055(19) | 0.0135(7) | 1.000(8) | 0.063(3) | 12c |
| O9 | O | 0.7819(19) | 0.5360(8) | 0.0280(7) | 1.000(8) | 0.063(3) | 12c |
| O10 | O | 0.2271(16) | 0.3209(18) | -0.1037(3) | 1.000(8) | 0.063(3) | 12c |
| O11 | O | 0.581(2) | 0.5528(10) | -0.0049(5) | 1.000(8) | 0.063(3) | 12c |
| O12 | O | 0.5845(10) | 0.4155(10) | -0.0833 | 1.000(8) | 0.063(3) | 6b |
| F1 | F | 0.89850 | 0.44920 | 0.08333 | 1.000(8) | 0.03800 | 6b |
| O31_1 | O | 0.32430 | 0.02530 | 0.11590 | 0.250(8) | 0.03800 | 12c |
| C32_1 | C | 0.23600 | -0.0830 | 0.09050 | 0.333(8) | 0.03800 | 12c |
| C33_1 | C | 0.25500 | -0.0310 | 0.04300 | 0.292(8) | 0.03800 | 12c |
| C34_1 | C | 0.27600 | 0.10200 | 0.05010 | 0.292(8) | 0.03800 | 12c |
| O35_1 | O | 0.15410 | 0.09100 | 0.05690 | 0.250(8) | 0.03800 | 12c |
| C36_1 | C | 0.18000 | 0.19800 | 0.08440 | 0.333(8) | 0.03800 | 12c |
| C37_1 | C | 0.27200 | 0.19800 | 0.11920 | 0.292(8) | 0.03800 | 12c |
| C38_1 | C | 0.34100 | 0.13900 | 0.09510 | 0.292(8) | 0.03800 | 12c |
| N39_1 | N | 0.37000 | -0.0220 | 0.02230 | 0.250(8) | 0.03800 | 12c |
| C40_1 | C | 0.40800 | -0.0020 | -0.0205 | 0.250(8) | 0.03800 | 12c |
| N41_1 | N | 0.52300 | 0.01900 | -0.0254 | 0.250(8) | 0.03800 | 12c |
| C42_1 | C | 0.56900 | 0.00600 | 0.01840 | 0.292(8) | 0.03800 | 12c |
| C43_1 | C | 0.47000 | -0.0180 | 0.04680 | 0.292(8) | 0.03800 | 12c |
| C44_1 | C | 0.32500 | -0.0010 | -0.0571 | 0.375(8) | 0.03800 | 12c |
| C45_1 | C | 0.59500 | 0.05500 | -0.0695 | 0.375(8) | 0.03800 | 12c |

| | | | | | | | |
|---|---|---|---|---|---|---|---|
| N46_1 | N | 0.36100 | 0.32800 | 0.13450 | 0.250(8) | 0.03800 | 12c |
| C47_1 | C | 0.40100 | 0.37700 | 0.17550 | 0.250(8) | 0.03800 | 12c |
| N48_1 | N | 0.49500 | 0.49400 | 0.17590 | 0.250(8) | 0.03800 | 12c |
| C49_1 | C | 0.53200 | 0.52800 | 0.12900 | 0.292(8) | 0.03800 | 12c |
| C50_1 | C | 0.44200 | 0.42400 | 0.10490 | 0.292(8) | 0.03800 | 12c |
| C51_1 | C | 0.34100 | 0.30000 | 0.21640 | 0.375(8) | 0.03800 | 12c |
| C52_1 | C | 0.57400 | 0.55500 | 0.21780 | 0.375(8) | 0.03800 | 12c |

Space group: $P6_122$ (178); $a = b = 12.2126(6)$ Å; $c = 30.1136(19)$ Å.
Residuals of the refinement: $R_{wp}$= 15.8; $R_{exp}$= 3.20; $R_B$= 6.49; $R_F$ = 5.33.
Due to the large complexity of the OSDA, the atomic parameters of the organic obtained by SCXRD for one of the crystals were used as is and not refined. Electron density of the H atoms has been added to that of the C atoms.

**Supplementary Table 8.**
Crystallographic parameters of calcined STW refined by PXRD.

| Site | Atom | *x* | *y* | *z* | Occupancy | Uiso | Wyckoff |
|---|---|---|---|---|---|---|---|
| Si1 | Si | 0.6955(4) | 0.3763(5) | 0.03398(15) | 0.477(7) | 0.0159(5) | 12c |
| Ge1 | Ge | 0.6955(4) | 0.3763(5) | 0.03398(15) | 0.523(7) | 0.0159(5) | 12c |
| Si2 | Si | 0.5271(5) | 0.4145(4) | 0.96602(13) | 0.291(6) | 0.0159(5) | 12c |
| Ge2 | Ge | 0.5271(5) | 0.4145(4) | 0.96602(13) | 0.709(6) | 0.0159(5) | 12c |
| Si3 | Si | 0.3021(4) | 0.4011(5) | 0.84946(16) | 0.390(8) | 0.0159(5) | 12c |
| Ge3 | Ge | 0.3021(4) | 0.4011(5) | 0.84946(16) | 0.610(8) | 0.0159(5) | 12c |
| Si4 | Si | 0.2456(5) | 0.3338(5) | 0.94921(18) | 0.674(8) | 0.0159(5) | 12c |
| Ge4 | Ge | 0.2456(5) | 0.3338(5) | 0.94921(18) | 0.326(8) | 0.0159(5) | 12c |
| Si5 | Si | 0.9621(5) | 0.1165(5) | 0.96626(15) | 0.843(7) | 0.0159(5) | 12c |
| Ge5 | Ge | 0.9621(5) | 0.1165(5) | 0.96626(15) | 0.157(7) | 0.0159(5) | 12c |
| O1 | O | 0.9125(11) | 0.00000 | 0.00000 | 1.00000 | 0.031(3) | 6a |
| O2 | O | 0.3416(7) | 0.5762(6) | 0.33284(20) | 1.00000 | 0.031(3) | 12c |
| O3 | O | 0.1191(4) | 0.2123(6) | 0.9707(2) | 1.00000 | 0.031(3) | 12c |
| O4 | O | 0.9306(4) | 0.0694(4) | 0.91670 | 1.00000 | 0.031(3) | 6b |
| O5 | O | 0.9026(8) | 0.2101(7) | 0.9766(2) | 1.00000 | 0.031(3) | 12c |
| O6 | O | 0.6433(8) | 0.3217(8) | 0.08330 | 1.00000 | 0.031(3) | 6b |
| O7 | O | 0.3707(4) | 0.3304(8) | 0.9668(3) | 1.00000 | 0.031(3) | 12c |
| O8 | O | 0.7708(6) | 0.3070(8) | 0.0147(2) | 1.00000 | 0.031(3) | 12c |
| O9 | O | 0.7888(7) | 0.5301(4) | 0.0360(3) | 1.00000 | 0.031(3) | 12c |
| O10 | O | 0.2398(8) | 0.3263(8) | 0.89626(15) | 1.00000 | 0.031(3) | 12c |
| O11 | O | 0.5741(9) | 0.5541(5) | 0.9871(2) | 1.00000 | 0.031(3) | 12c |
| O12 | O | 0.5776(4) | 0.4224(4) | 0.91670 | 1.00000 | 0.031(3) | 6b |

Space group: $P6_122$ (178); $a = b$ = 12.1528(12) Å; $c$ = 30.128(3) Å.
Residuals of the refinement: $R_{wp}$= 12.0; $R_{exp}$= 6.63; $R_B$= 5.19; $R_F$ = 3.18.

**Supplementary Table 9**

Catalytic activity of Al containing-S-STW zeolite (sample 2-STW-25) for the enantioselective 1,2-epoxyalkane ring opening reaction.

| **Catalyst** | **epoxide** | **Conversion (%)** | **Selectivity to A (%)** | **Selectivity to B (%)** |
|---|---|---|---|---|
| Al-S-STW | 1,2,-epoxibutane | 6 | 67 (e.e.: 0) | 33 (e.e.: 2.5) |
| Al-S-STW | 1,2-epoxihexane | 2 | 49 (e.e.: 4) | 51 (e.e.: 3.6) |
| FAU-15 | 1,2-epoxybutane | 94 | 46 (e.e.: 0) | 54 (e.e.: 0) |

Reaction temperature: 25ºC, 7 mmol epoxide, 14 mmol i-propanol, 30 mg Al-S-STW, reaction time: 48h

**Product A** **Product B**

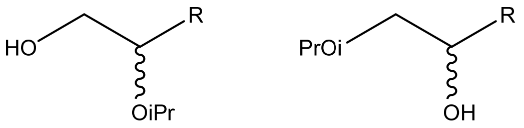


This table shows that the achieved conversion for 1,2-epoxyhexane ring-opening was 2%, with an approximate 1:2 ratio of product A to product B, with an enantiomeric excess (e.e.) ranging between 4 to 3.5% in the resultant products. When evaluating shorter alkyl chain epoxides, such as 1,2-epoxybutane, a higher conversion rate of 6% was achieved, albeit with e.e. in the products ranging from 1.5 to 2.5%.

When compared with the results obtained using another zeolite with a larger pore size, such as a commercial faujasite with Si/Al = 15, it is observed a very significant increase in conversion, reaching nearly quantitative values, with no e.e. This supports the hypothesis that poor diffusion of reactants through the smaller pores of the S-STW material limits reaction activity.

This limitation can be attributed to the very large crystal size of the Al-S-STW catalyst, hampering the diffusion of reactants and/or products through the helicoidal pores during the reaction, and therefore the observed low activity is mostly occurring at the external surface of the crystals of zeolite STW.
